# Oleoylethanolamide, an endogenous PPAR-α ligand, attenuates liver fibrosis targeting hepatic stellate cells

**DOI:** 10.18632/oncotarget.6466

**Published:** 2015-12-04

**Authors:** Ling Chen, Long Li, Junde Chen, Lei Li, Zihan Zheng, Jie Ren, Yan Qiu

**Affiliations:** ^1^ Department of Medical Sciences, Medical College, Xiamen University, Xiamen, Fujian, China; ^2^ Marine Biological Resource Comprehensive Utilization Engineering Research Center of the State Oceanic Administration, The Third Institute of Oceanography of the State Oceanic Administration, Xiamen, Fujian, China; ^3^ Xiamen Diabetes Institute, The First Affiliated Hospital of Xiamen University, Xiamen, Fujian, China; ^4^ Clinical Research Institute, The First Affiliated Hospital, University of South China, Hengyang, Hunan, China; ^5^ College of Arts and Sciences, University of North Carolina at Chapel Hill, Chapel Hill, North Carolina, USA

**Keywords:** liver fibrosis, OEA, PPAR-α, TGF-β1, hepatic stellate cells, Pathology Section

## Abstract

Oleoylethanolamide (OEA), an endocannabinoid-like molecule, was revealed to modulate lipid metabolism through a peroxisome proliferator-activated receptor-*α* (PPAR-*α*) mediated mechanism. In present study, we further investigated the activities and mechanisms of OEA in ameliorating hepatic fibrosis in Sv/129 mice induced by a methionine choline-deficient (MCD) diet or thioacetamide (TAA) treatment. Liver fibrosis development was assessed by Hematoxylin-eosin and Sirius red staining. Treatment with OEA (5 mg/kg/day, intraperitoneal injection, i.p.) significantly attenuated the progress of liver fibrosis in both two experimental animal models by blocking the activation of hepatic stellate cells (HSCs). Gene expression analysis of hepatic tissues indicated that OEA inhibited the expression of *α*-smooth muscle action (*α*-SMA) and collagen matrix, fibrosis markers, and genes involved in inflammation and extracellular matrix remodeling. *In vitro* studies showed that OEA inhibited transforming growth factor *β*1-stimulated HSCs activation through suppressing Smad2/3 phosphorylation, *α*-SMA expression and myofibroblast transformation. These improvements could not be observed in PPAR-*α* knockout mice models with OEA administration, which suggested all the anti-fibrotic effects of OEA *in vivo* and *in vitro* were mediated by PPAR-*α* activation. Collectively, our results suggested that OEA exerted a pharmacological effect on modulating hepatic fibrosis development through the inhibition of HSCs activation in liver and therefore may be a potential therapeutic agent for liver fibrosis.

## INTRODUCTION

Liver fibrosis is a common wound healing response that may be mounted in response to chronic or repeated liver injury. Various etiological factors, including chronic hepatitis virus infection, nonalcoholic steatohepatitis (NASH), genetic mutations, and cholestatic diseases can drive fibrosis. About 25-40% of liver fibrosis cases lead to cirrhosis. The same pathological processes behind fibrosis have also been linked with hepatocellular carcinoma (HCC) [1, 2]. This pathological process is characterized by excessive production and deposition of proteins of the extracellular matrix (ECM). Hepatic stellate cells (HSCs) are recognized as the main producers of matrix components in the liver, and play a critical role in regulating the production and secretion of the ECM. In normal livers, HSCs stay in a quiescent state, mainly serving to store vitamin A. These quiescent HSCs may trans-differentiate following liver injury however, changing into highly-proliferative myofibroblast-like cells that are characterized by the expression of *α*-smooth muscle actin (*α*-SMA), and excessive production of type I collagen (Col1a) and type III collagen (Col3a), which are critical components of the ECM. The trans-differentiation process of HSCs into myofibroblasts is mainly regulated by canonical TGF-*β*1/Smad signaling. Suppression of HSC activation has been proposed as therapeutic strategy for the treatment and prevention of liver fibrosis, and novel methods for achieving this end are still being sought [3-5].

Peroxisome proliferator-activated receptors (PPARs) are members of the nuclear hormone receptor family of ligand-activated transcription factors, consisting of three different isoforms; PPAR-α, PPAR-β/δ and PPAR-γ. PPARs play important roles in controlling many physiological processes, including inflammation and fibrogenesis. Due to their unique tissue distribution patterns, the roles of the three PPARs are at least spatially, if not always functionally, distinct. PPAR-α is predominantly present in the liver [6]. Previous studies have demonstrated that PPAR-*α* plays a critical role in modulation of energy balance and regulation of hepatic lipid metabolism [7, 8]. PPAR-*α* serves as a key signal transducer in these pathways, acting downstream of sensors such as AMP kinase and aldose reductase. A synthetic PPAR-α ligand, fenofibrate, functions to reduce serum triglyceride levels, and is clinically used to ameliorate plasma lipid disorders at risk of cardiovascular disease [9]. Some clinical studies have shown that fenofibrate also plays a key role in maintaining the normal liver function and improving insulin resistance in NAFLD patients [10]. In addition, several observations have indicated that PPAR-*α* might also play a pivotal role in the molecular control of fibrogenesis. Previous manuscripts have reported that PPAR-*α* agonists Wy-14643 and fenofibrate may prevent the development of hepatic fibrosis in the rat thioacetamide (TAA) and methionine choline-deficient (MCD) models of liver fibrosis [11, 12]. PPAR-*α* has also been shown to be significantly involved with inflammation, as activation of PPAR-*α* protects against hepatic ischemia reperfusion injury in mice [13]. Newer studies add to a growing body of evidence that PPAR-*α* could be promising a therapeutic target in physiological and pathological processes involved in liver diseases [14].

Oleoylethanolamide (OEA), a high affinity endogenous ligand of PPAR-*α*, has been identified to play an important role in the treatment of obesity and arteriosclerosis [15-17]. In contrast to Wy-14643 and fenofibrate, OEA is not a specific ligand for PPAR-*α,* as it can act via other receptors such as the vanilloid receptor (TRPV1) and GPR119, allowing it to have diverse physiological functions [18, 19]. The role of OEA in liver fibrosis has not been well elucidated. In this study, we investigated the effect of OEA treatment on the progression of liver fibrosis in chronic MCD diet-induced and TAA-induced experimental models. We demonstrate that OEA acts through a PPAR-*α* dependent mechanism to ameliorate liver fibrosis, and observe that TGF-*β*1–mediated HSC activation is also involved.

## RESULTS

### OEA reverses MCD diet-induced steatohepatitis, liver fibrosis, and leukocyte infiltration

**Figure 1:**
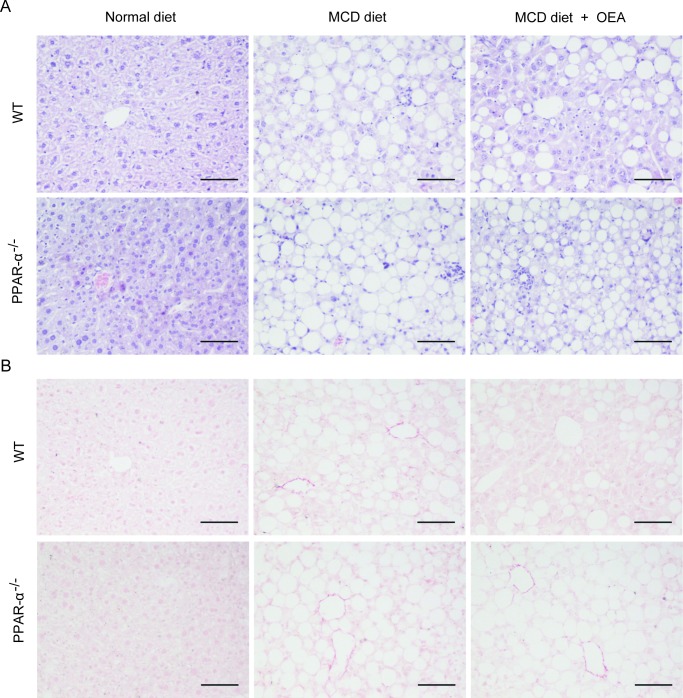
OEA improved liver histology in MCD diet-induced fibrosis mice via PPAR-*α* A. Hematoxylin-eosin (HE) staining of liver sections in wild-type (WT) mice and PPAR-α knockout mice fed with normal diet, MCD diet, MCD diet combined with OEA administration (5 mg/kg/day, i.p.). **B**. Sirius red staining of liver sections in wild-type (WT) mice and PPAR-α knockout mice fed with normal diet, MCD diet, MCD diet combined with OEA administration (5 mg/kg/day, i.p.). Scale bars: 100 *μ*m. *n* = 6-8 in each group.

**Figure 2:**
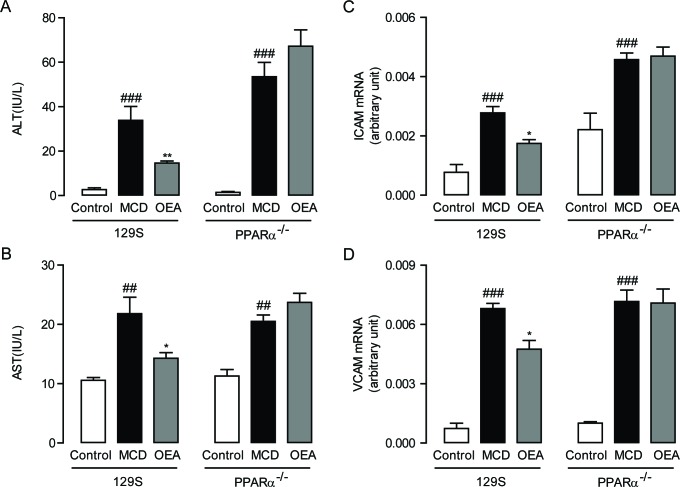
OEA alleviated liver injury and inflammation in MCD diet-induced fibrosis mice through PPAR-*α* **A**.-**B**. Effect of OEA on plasma ALT (A) and AST (B) levels in wild-type (WT) mice and PPAR-*α* knockout mice fed with normal diet, MCD diet, MCD diet combined with OEA administration (5 mg/kg/day, i.p.). **C**.-**D**. Effect of OEA on liver mRNA expression levels of ICAM (C) and VCAM (D) in wild-type (WT) mice and PPAR-*α* knockout mice fed with normal diet, MCD diet, MCD diet combined with OEA administration (5 mg/kg/day, i.p.). Data are shown as means ± s.e.m.; *n* = 6-8 in each group. ^##^ *P* < 0.01, ^###^ *P* < 0.001, ^*^ *P* < 0.05, ^**^ *P* < 0.01.

In order to evaluate the anti-fibrosis role of OEA *in vivo*, we first established a liver fibrosis model using MCD diet-induced Sv/129 mice. Histological analysis via H&E, Sirius red, and Oil red O staining staining was used to gauge the extent of liver injury induced by the MCD diet. After 8 weeks MCD diet feeding, both PPAR-*α* knockout mice and WT mice developed moderate steatosis and severe hepatocyte ballooning (Figure 1A, S1A, B). Significant deposition of fibrillary collagens was also detected in the livers of both mice types (Figure 1B), along with significantly elevated serum levels of ALT (*P* < 0.001, *P* < 0.001), AST (*P* < 0.01, *P* < 0.01) (Figure 2A, 2B), and liver levels of TG (*P* < 0.001, *P* < 0.01) (Figure S1C). In contrast, livers from OEA administration groups exhibited ameliorated steatosis and reduced hepatocyte ballooning (Figures 1A, S1A, S1B), along with the improvements observable via Sirius-red staining (Figure 1B). Interestingly, chemical analysis of serum and hepatic composition indicate that OEA treatment partially prevented the increases of ALT, AST and TG levels observed in WT mice given MCD diet (*P* < 0.01, *P* < 0.05, *P* < 0.05, respectively), but did not attenuate the increase in PPAR-*α* knockout groups (Figure 2A, 2B and Figure S1C).

In order to further confirm that OEA treatment could bring about sustained amelioration of liver fibrosis, we next assessed the impact that OEA treatment had on immune cells. Histological analysis demonstrated that the MCD diet led to significant increases in the number of leukocytes in the livers of both WT mice and PPAR-*α* knockout mice, but that treatment with OEA reduced this recruitment in WT mice only. (Figure 1A). OEA treatment also led to significant reductions in the mRNA expression of the adhesion molecules ICAM and VCAM, two key proteins responsible for mediating the recruitment of immune cells to sites of liver injury in WT MCD-fed mice, but could not suppress their expression in the absence of PPAR-*α* (Figure 2C, 2D). From these results, it seems clear that OEA can play a potent role in repressing liver damage via a PPAR- *α* dependent mechanism.

### OEA suppresses expression of hepatic pro-fibrogenic and pro-remodeling genes

**Figure 3:**
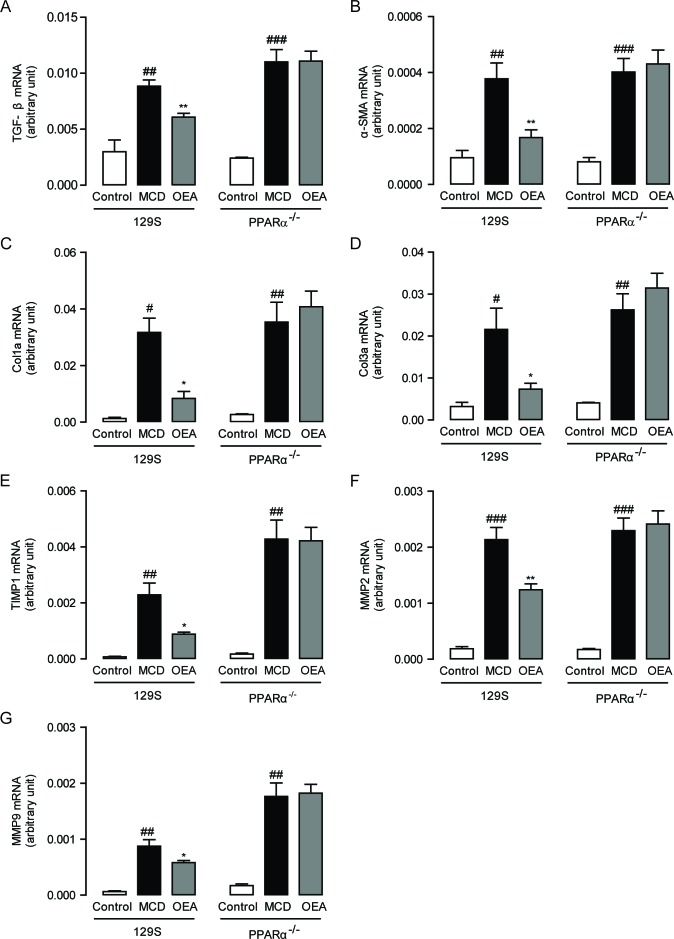
OEA modulated hepatic fibrotic genes expression in MCD diet-induced fibrosis mice by PPAR-α The effect of OEA on liver mRNA levels of TGF-β **A**., α-SMA **B**., Col1a **C**., Col3a **D**., TIMP1 **E**., MMP-2 **F**., and MMP-9 **G**. in wild-type (WT) mice and PPAR-*α* knockout mice fed with normal diet, MCD diet, MCD diet combined with OEA administration (5 mg/kg/day, i.p.). Data are shown as means ± s.e.m.; *n* = 6-8 in each group. ^#^ *P* < 0.05, ^##^ *P* < 0.01, ^###^ *P* < 0.001, ^*^ *P* < 0.05, ^**^ *P* < 0.01.

To further determine the mechanisms by which OEA protects against MCD diet-induced liver fibrosis, we then assessed the hepatic mRNA levels of several relevant genes through qPCR. As shown in Figure 3A-3D, expression of TGF-*β*1, *α*-SMA, Col1a and Col3a significantly increased in both WT mice and PPAR-*α* knockout mice once fed the MCD diet. These increases were all substantially reversed by treatment with OEA in WT mice, but not in PPAR-*α* knockout mice. It is also noteworthy that the mRNA and protein expression levels of PPAR-*α* were reduced with MCD treatment in WT mice, but that these changes were reversed by OEA treatment (Figure S2A-S2D). These findings indicate that the anti-fibrogenic properties displayed by OEA are PPAR-*α* dependent.

Besides observing changes in the expression of pro-fibrogenic genes following OEA therapy, we also investigated for possible alterations in the expression of genes responsible for managing ECM remodeling. After all, it has been suggested that ECM remodeling regulates the development of fibrosis and matrix degradation that occurs as a consequence of changes in the balance of matrix metalloproteinases (MMPs) and tissue inhibitors of metalloproteinases (TIMPs) [20]. In both WT and PPAR-*α* knockout mice, the MCD group exhibited significantly upregulated levels of TIMP1, MMP2, and MMP9 (Figure 3E-3G). This type of expression pattern is consistent with the expected compensation pattern mounted by hepatocytes in response to collagen deposition. Treatment with OEA significantly suppressed the induction of these remodeling proteins in WT mice (Figure 3E-3G).

### OEA attenuates TAA-induced liver fibrosis in mice through PPAR-α

**Figure 4:**
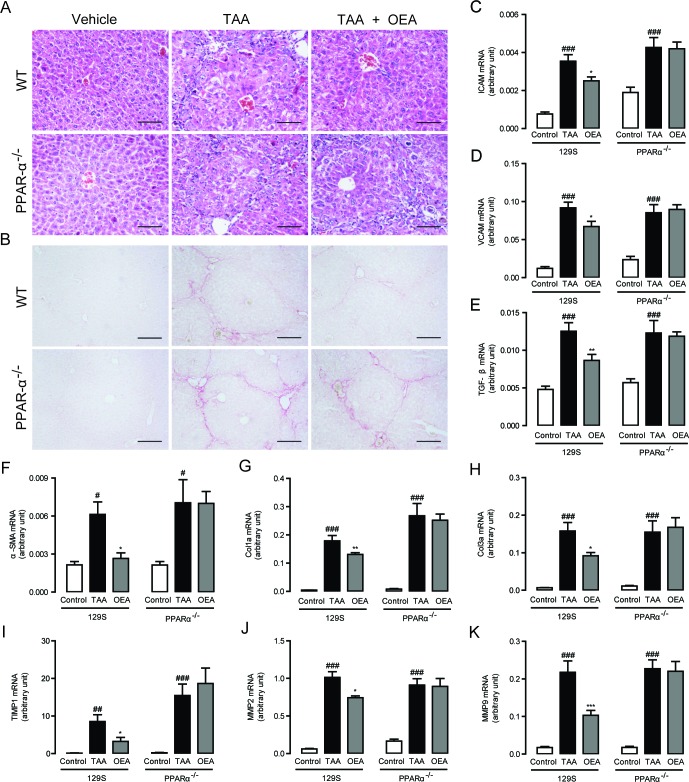
Anti-fibrotic effects of OEA in TAA-induced fibrosis mice were mediated by PPAR-*α* activation **A**.-**B**. Hematoxylin-eosin (HE) and Sirius red staining of liver sections. **C**.-**K**. Hepatic mRNA levels of ICAM, VCAM, TGF-*β*, *α*-SMA, Col1a, Col3a, TIMP1, MMP-2, and MMP-9 were determined by quantitative real-time PCR analysis. Results from wild-type (WT) mice and PPAR-*α* knockout mice with saline treatment, TAA treatment (160 mg/kg, i.p.), TAA treatment combined with OEA administration (5 mg/kg/day, i.p.). Data are shown as means ± s.e.m.; *n* = 6-8 in each group. ^##^ *P* < 0.01, ^###^ *P* < 0.001, ^*^ *P* < 0.05, ^**^ *P* < 0.01, ^***^ *P* < 0.001. Scale bars: 100 μm (A), 200μm (B).

In order to further confirm the ability of OEA to reverse liver fibrosis, we further tested the effect of OEA on TAA-induced hepatic fibrogenesis as a second experimental model. Significant liver fibrosis was observed in both PPAR-*α* knockout mice and WT mice in response to TAA injection. Similar to the results in MCD diet-induced liver fibrosis model, OEA remarkably prevented the progression of TAA-induced hepatic fibrosis in WT mice, but not in PPAR-*α* knockout mice, as identified by H&E staining and Sirius Red staining (Figure 4A, 4B). These decreases were associated with significant decreases in ICAM and VCAM mRNA expression (Figure 4C, 4D), a marked decrease in TGF-*β* and *α*-SMA mRNA expression (Figure 4E, 4F), and a significant reduction in Col1a and Col3a mRNA expression (Figure 4G, 4H) after OEA administration in TAA-treated WT mice. The same reduction pattern was also observed in the mRNA expression levels of TIMP1, MMP-2 and MMP-9 after OEA treatment in the TAA-treated WT mice (Figure 4I-4K). By contrast, PPAR-*α* knockout mice were completely insensitive to OEA treatment. Take together with our previous results, it seems clear then that OEA can generally ameliorate liver fibrosis via a PPAR-*α*-dependent mechanism.

### OEA suppresses TGF-*β*1 induced HSC activation *in vitro*

**Figure 5:**
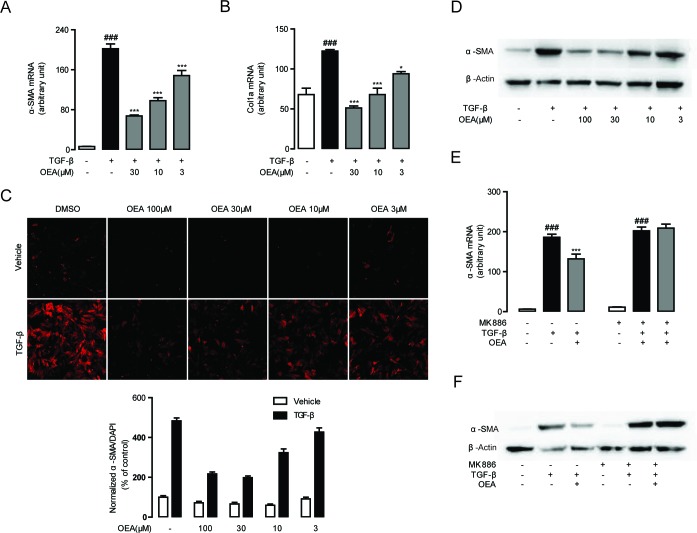
OEA suppressed TGF-*β*1 induced HSCs activation *in vitro* via PPAR-*α* **A**.-**B**. CFSC cells were treated with OEA (30 μM, 10 μM, 3 μM) followed by TGF-*β*1 (5 ng/mL) for 48 h, mRNA expression levels of *α*-SMA (A) and Col1a (B) were analyzed by real-time PCR. **C**.-**D**. Immunofluorescence staining (C) and western blot (D) were performed to detect protein expression levels of *α*-SMA. **E**.-**F**. CFSC cells were treated with OEA (10 μM) followed by TGF-*β*1 (5 ng/mL) for 48 h with or without MK886 (10 μM) treatment. The *α*-SMA mRNA expression levels were measured by real-time PCR (E). Protein expression levels of *α*-SMA were assessed by Western Blot (F). Data are shown as means ± s.e.m. of three independent experiments each performed in duplicate. ^###^ *P* < 0.001, ^*^ *P* < 0.05, ^***^ *P* < 0.001.

**Figure 6:**
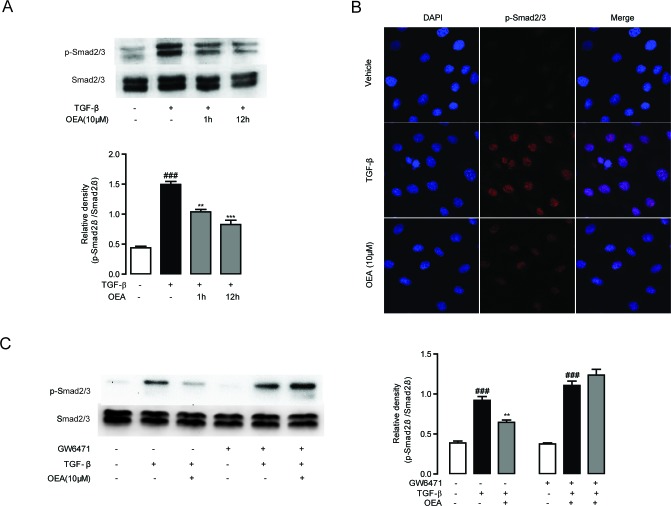
Inhibitory effect of OEA on TGF-*β*1-dependent Smad2/3 phosphorylation *in vitro* was mediated by PPAR-α **A**. CFSC cells were treated with TGF-*β*1 (5 ng/mL) for 30 min after 1 h or 12 h OEA (10 μM) treatment, Smad2/3 phosphorylation was analyzed by Western Blot. **B**. CFSC cells were treated with TGF-*β*1 (5 ng/mL) for 30 min after 1 h OEA (10 μM) treatment, phosphorylation of Smad2/3 was analyzed by Immunofluorescence staining. **C**. CFSC cells were treated with TGF-*β*1 (5 ng/mL) for 30 min after 1 h OEA (10 μM) treatment in the absence or presence of GW6471 (10 μM). Data are shown as means ± s.e.m of three independent experiments. ^###^ *P* < 0.001, ^**^ *P* < 0.01, ^***^ *P* < 0.001.

Following our observations that OEA attenuation of fibrosis is dependent on PPAR-α, we next sought to characterize the molecular mechanisms behind the attenuation. We first explored for changes in TGF-*β*1 signaling, since that pathway is essential for HSC activation and liver fibrosis. Numerous lines of evidence have shown that TGF-*β*1 treatment upregulates the expression of several pro-fibrogenic genes such as *α*-SMA and Col1a in quiescent fibroblasts [1, 3]. To assess the impact of OEA on HSCs activation, the expression levels of *α*-SMA and Col1a in TGF-*β*1-stimulated HSCs were examined by qPCR. The mRNA levels of *α*-SMA and Col1a were markedly induced in the group of CFSC cells with TGF-*β*1 (5 ng/mL) stimulation for 48h, while the mRNA levels were suppressed when treated with OEA in a dose-dependent manner (Figure 5A, 5B). Immunofluorescence and western blot results showed that OEA treatment dose-dependently inhibited the protein expression of *α*-SMA (Figure 5C, 5D), the marker of HSC activation. As shown in Figure 5E, 5F, the inhibitory effects of OEA on HSCs activation were completely blocked by PPAR-*α* antagonist MK886 (10 μM). Moreover, the mRNA and protein expression levels of PPAR-*α* were down-regulated with TGF-*β*1 stimulation, while OEA treatment restored these changes in dose-dependent manner (Figure S2E, S2F). In addition, the phosphorylation of Smad 2/3 was upregulated in the presence of TGF-*β*1 stimulation, consistent with the observed effects on HSC activation, while OEA (10 μM) reduced the phosphorylation of Smad2/3 in CFSC simulated with TGF-*β*1. The altered pattern of phosphorylation was also effectively blocked by the PPAR-*α* antagonist GW6471 (10 μM), further demonstrating the PPAR-*α* dependence of the phenomenon (Figure 6).

Having verified that PPAR-*α* impacts the TGF-*β*1/Smad pathway, we then examined the pathway to better understand the molecular mechanisms by which it may influence signaling. Bioinformatics analysis revealed a putative binding site for the PPAR-*α*­-RXR-*α* heterodimer on the TGF-*β*1 promotor. A dual-luciferase reporter assay showed that PPAR-*α* overexpression slightly enhanced the TGF-*β*1 promoter activity (Figure S3A), while OEA treatment (30, 10, 3 μM) had no effect (Figure S3B). As such, it seems that OEA does not directly repress TGF-*β*1 promotor activation by increasing PPAR-*α* activity or expression. We also detected the genes involved into the formation and degradation of phospho-Smad2/3. The mRNA expression of TGFBR1, Smad4 and PPM1A all increased after OEA treatment, while that of TGFBR2 and Smad7 was not affected (Figure S4). As such, it seems that the influence of OEA-activated PPAR-*α* on pSmad2/3 occurs through other mechanisms.

## DISCUSSION

The critical roles played by endogenous ligands in mediating cellular signaling is becoming increasingly appreciated, with many metabolites now being understood to have potent effects on cellular behavior. Our present study demonstrates that the endogenous PPAR-*α* ligand, OEA, can significantly suppress the pro-fibrotic cytokine TGF-*β*1 negatively regulate genes in the TGF-*β*1 signaling pathway (*α*-SMA, collagen 1a, and collagen 3a) in mice models of hepatic fibrosis. We also show that OEA treatment inhibited the increase of serum ALT, AST, and hepatic TG levels, downregulated the adhesion molecules ICAM and VCAM, and reduced the gene expression of matrix remodeling enzymes TIMP1, MMP2, and MMP9 to help slow the pathogenesis of fibrosis. These improvements as a result of OEA treatment were lost in PPAR-*α* knockout mice models, showing that the effects were almost completely dependent on PPAR-*α* function. Although similar anti-fibrogenic properties of synthetic PPAR-*α* agonists, Wy-14643 and fenofibrate, have been reported in TAA, MCD, CCl_4_ and ethanol-induced liver fibrosis [11, 12, 21], previous studies had not elucidated the mechanisms behind the response. In addition, off-target roles of these synthetic ligands may prevent them from being fully effective. For instance, Wy-14643 caused regression of hepatic fibrosis in MCD dietary feeding models, but failed to reduce the increased TGF*-β*1 mRNA expression [11], making it unclear as to if it could be truly effective at longer-term amelioration.

Liver fibrosis is presently understood to be a process of sterile inflammation caused by a vicious cycle of hepatic damage driving HSC activation and worsening hepatic damage [12]. Hepatic injury caused by MCD diet and TAA increases ER stress and induces MAPK/ERK activation, which is considered as an indispensable step for the overexpression of TGF-*β*1 mRNA [22]. As a critical survival factor for HSCs, TGF-*β*1 plays an important role in triggering and maintaining the vicious cycle. In the canonical TGF-*β*1 signal transduction pathway, binding of TGF-*β*1 to TGF-*β*1 type I/II receptor induces phosphorylation of Smad2/3, which is released into the cytosol and results in dimerization with Smad4. The heterodimer then translocates into the nucleus and regulates transcription [23]. So far, the effects and molecular mechanisms of PPAR-*α* in TGF-*β*/Smad signaling remains elusive.

It is also possible that different molecular mechanisms are responsible for the effects of PPAR-*α* in different cell types. After all, it has been reported that Wy14643 could not reduce phospho-Smad2/3 induced by TGF-*β* in rat chondrocytes [24], while, in another paper, Wy14643 and clofibrate were reported to significantly decrease phosphorylation of Smad2 and Smad3 in 10T1/2 cells [25]. In the present study, we demonstrate that phosphorylation of Smad2/3 was suppressed by OEA in CFSC cells, and that these effects were reversed by blockage of PPAR-*α* activation. These results suggested that PPAR-*α* may antagonize the transcriptional effects induced by TGF-*β*1. Previous reports have shown that a physical interaction between ligand-activated PPAR-*α* and Smad4 suppressed TGF-*β*1-induced integrin transcription, but a direct binding between them could not be detected [26]. Our qRT-PCR results indicate that OEA did not reduce the expression of several genes involved in the formation of phospho-Smad2/3, (TGFBR1/2 and Smad 4, as seen in Figure S4B, S4C, S4F), while it increased the mRNA expression of PPM1A (Figure S4D), a phosphatase that dephosphorylated and promoted nuclear export of activated Smad2/3 to terminate TGF-*β* signaling [23, 27]. It is also possible that the nuclear translocation step is affected, and raising the possibility that PPAR-*α* might act as a gatekeeper of sorts for transcription regulation given its positioning.

TGF-*β*1 signaling drives downstream HSC expression of α-SMA and collagen type 1a/3a, which are key markers for fibrosis. Overexpressed TGF-*β*1 has also been shown to increase ROS levels, which impairs PPAR-*α* expression [12] and inhibits PPAR-*α* activity [28] in hepatocytes. In the present study, TGF-*β*1 repressed the mRNA expression of PPAR-*α*, which could be reversed by SB-431542, a TGF-*β* pathway inhibitor (Figure S5). At the same time, PPAR-*α* ligands have been identified to reduce ROS levels to reverse the PPAR-*α* reduction and subsequently alleviate fibrosis [12]. Administration of OEA induced a partial down-regulation of the TGF-*β*1 mRNA expression (Figures 3A, 4E) by rescuing PPAR-*α* mRNA (Figure S2) and activating JNK and p38 MAPK [29] to inhibit the activation of HSCs, although PPAR-*α* overexpression and OEA did not repress TGF-*β* promotor activity directly (Figure S3). OEA treatment also led to the suppression of vital ECM and matrix remodeling genes, which contribute greatly to the vicious cycle of worsening damage. Taken together, these data demonstrate that endogenous PPAR-*α* agonist OEA effectively suppressed activation of HSCs and liver fibrosis through effects on TGF-*β*1. Future work to elucidate mechanisms to increase OEA expression may help further increase its therapeutic potential.

## MATERIALS AND METHODS

### Reagents

OEA was synthesized in our lab as previously described [30]. Recombinant Human TGF-*β*1 was from R&D Systems (Shanghai, China). Dimethyl sulfoxide (DMSO), TAA and all other chemicals were obtained from Sigma–Aldrich (Shanghai, China) if not mentioned otherwise. Dulbecco's modified Eagle's medium (DMEM) and fetal bovine serum (FBS) were purchased from Invitrogen (Shanghai, China). MK886, a PPAR-*α* inhibitor, was purchased from Cayman Chemical (Michigan, USA). GW6471, a PPAR-*α* antagonist, was purchased from R&D Systems (Shanghai, China). Alanine transaminase (ALT) and aspartate transaminase (AST) commercial assay kits were purchased from Nanjing Jiancheng Bioengineering Institute (Nanjing, China).

The chemical constituents were dissolved in saline supplemented with 5% polyethylene glycol 400 (PEG400) and 5% Tween-80 for the *in vivo* studies. For the *in vitro* studies, OEA was dissolved in DMSO to a series concentration of 100-3 mmol/L (stock solution) and then diluted in the culture medium to 100-3 μmol/L.

### Animals and treatments

The Sv/129 mice were purchased from the Model Animal Research Center (Nanjing, China). The PPAR-α knockout mice were obtained from Jackson Laboratory (Bar Harbor, ME, USA). All procedures were in performed in compliance with the guidelines for animal care and use and were approved by the Committee for Animal Research at Xiamen University. The mice were maintained in a room with controlled temperature (21–23°C), humidity (55–60%) and lighting (12 h light/dark cycles) and given water ad libitum. Mice were randomly divided for MCD and TAA experiments. In the MCD-diet feeding experiment, wild-type Sv/129 mice and PPAR-α knockout mice were each divided into three groups (*n* = 8 /group): (i) control group received normal diet; (ii) fed with MCD diet and injected with the vehicle (5% Tween-80 + 5% PEG400 + 90% saline, 5 mL/kg/day, 8 weeks, intraperitoneal injection, i.p.); (iii) fed with MCD diet along with OEA administration (5 mg/kg/day; 8 weeks, i.p.). In another set of experiment, all the wild-type mice and PPAR-α knockout mice were given standard chow diet, and were randomly separated into three groups: the control group was not administrated TAA or OEA but was injected with the saline; the TAA group was injected with TAA (160 mg/kg, three times per week, 6 weeks, dissolved in saline, i.p.) plus the corresponding vehicle; the OEA group was both injected with TAA and OEA (5 mg/kg/day; 6 weeks, i.p.).

### Liver histological studies

Fresh liver biopsy specimen fixed in the 10% neutral-buffered formalin for 3 days and then embedded with paraffin for histological examinations. Sections of 5 μm were cut by a Leica SM2010 R Sliding microtome (Shanghai, China) and stained with hematoxylin-eosin (H&E) or Sirius red to assess liver damage and fibrosis development. To determine the level of lipid accumulation in liver, frozen sections of formalin-fixed liver were stained with Oil red O. Stained areas were viewed and imaged through standard microscopy (Nikon, Shanghai, China).

### Plasma and hepatic biochemistry assays

For testing liver function, plasma aspartate transaminase (ALT), alanine transaminase (AST) and hepatic triglyceride (TG) concentration were determined by a Thermo Scientific Multiskan GO Microplate Spectrophotometer with commercial kits.

### Cell culture

CFSC, HSC cell lines were first obtained from cirrhotic rat liver, and have a similar phenotype to that of early passage primary HSCs. CFSC cells (kind gift from Dr. Chenggang Zhu) were cultured in Dulbecco's modified Eagle's medium (DMEM) supplemented with 10% fetal bovine serum (FBS) and 1% penicillin/streptomycin. All cells were cultured in 6-well culture plates under 37°C and 5% CO­_2_ in an incubator. The medium was replaced every two days, and the cells were harvested and diluted at a ratio of 1:3 twice a week. In experiments, HSCs were pretreated with the experimental concentration of OEA before stimulation with 5 ng/mL TGF-*β*1.

### RNA isolation and cDNA synthesis

Total RNA from liver tissues and cells was extracted using the TRIzol^TM^ isolation reagent (Invitrogen) according to the protocol provided by the manufacturer. cDNA was synthesized from total RNA using a ReverTra Ace® qPCR RT kit (Toyobo, Shanghai, China) according to the manufacturer's instructions.

### Real-time PCR

Quantification of mRNA was carried out on an Applied Biosystems 7300 real-time polymerase chain reaction (PCR) system using SYBR® Premix Ex Taq™ II (Takara, Dalian, China). The quantitative values of mRNA were normalized relative to the levels of GAPDH or 18s mRNA.

### Immunofluorescence analysis

Cells were seeded in 24-well plates and fixed with 4% paraformaldehyde for 15 min at room temperature (RT). after blocking with 2% bovine serum albumin (BSA) in PBS for 1 h at RT, the cells were incubated with primary Mouse anti-*α*-SMA (1:100, Sigma-Aldrich) or Rabbit anti-p-Smad2/3 (1:100, Cell Signalling Technology) antibodies diluted with 1% BSA overnight at 4°C, followed by three washes with PBS and incubation for 2 h with secondary antibodies (donkey anti-mouse IgG conjugated with Alexa Fluor 594 or donkey anti-rabbit IgG conjugated with Alexa Fluor 594) diluted with 1% BSA for 1 h at RT. Cell nuclei were stained with 4,6-diamidino-2-phenylindole (DAPI). Images were captured using a laser scanning confocal microscope (FluoView 1000; Olympus, Tokyo, Japan).

### Western blot analysis

Western blot analysis was conducted as previously reports [31]. The target protein was detected using primary antibodies as follows: Mouse anti-*α*-SMA (1:500, Sigma-Aldrich), Rabbit anti-p-Smad2/3 (1:1000, Cell Signaling Technology), Rabbit anti-Smad2/3 (1:1000, Cell Signaling Technology), Mouse anti-PPAR-*α* (1:1000, Abcam) and Mouse anti-*β*-actin (1:5000, Proteintech).

### Statistical analysis

All statistical analyses were performed with GraphPad Prism version 5.01 for Windows. Results were expressed as the mean ± s.e.m. Statistical analysis was performed via one-way analysis of variance (ANOVA) followed by Dunnett's test for multiple comparisons. *P* < 0.05 was considered statistically significant.

## SUPPLEMENTARY MATERIAL FIGURES


