# Supplementary material for: Oleoylethanolamide, an endogenous PPAR-α ligand, attenuates liver fibrosis targeting hepatic stellate cells
Source: Oncotarget. 2015 Dec 4;6(40):42530–40. doi: 10.18632/oncotarget.6466 (PMC4767450; doi:10.18632/oncotarget.6466)
Supplement: Supplementary file 1 [file oncotarget-06-42530-s001.pdf]

Oleoylethanolamide, an endogenous PPAR-α ligand, attenuates liver fibrosis targeting hepatic stellate cells

Supplementary Material

Primer sequences for Real-time PCR

Primer sequences were synthesized as follows: Rat Col1a, 5'-CCC ACC GGC CCT ACT G-3' (forward), 5'-GAC CAG CTT CAC CCT TAG CA-3' (reverse); Rat *α*-SMA, 5'-AGC TCT GGT GTG TGA CAA TGG-3' (forward), 5'-GGA GCA TCA TCA CCA GCA AAG-3' (reverse); Rat PPAR-*α*, 5'-AAT CCA CGA AGC CTA CCT GA-3'(forward), 5'-GTC TTC TCA GCC ATG CAC AA-3' (reverse); Rat GAPDH, 5'-ACC ACG AGA AAT ATG ACA ACT CCC-3' (forward), 5'-CCA AAG TTG TCA TGG ATG ACC-3' (reverse); Mouse Col1a, 5'-ACG GCT GCA CGA GTC ACA C-3' (forward), 5'-GGC AGG CGG GAG GTC TT-3'(reverse); Mouse *α*-SMA, 5'-CAG GCA TGG ATG GCA TCA ATC AC-3' (forward), 5'-ACT CTA GCT GTG AAG TCA GTG TCG-3'(reverse); Mouse TGF-*β*1, 5'-TGA CGT CAC TGG AGT TGT ACG G-3' (forward), 5'-GGT TCA TGT CAT GGA TGG TGC-3' (reverse); Mouse Col3a, 5'-GTT CTA GAG GAT GGC TGT ACT AAA CAC A-3' (forward), 5'-TTG CCT TGC GTG TTT GAT ATT C-3' (reverse); Mouse ICAM, 5'-CGC TGT GCT TTG AGA ACT GT-3' (forward), 5'-GGT GAG GTC CTT GCC TAC TT-3' (reverse); Mouse VCAM, 5'-GAA CCC AAA CAG AGG CAG AG-3' (forward), 5'-GGT ATC CCA TCA CTT GAG CAG-3' (reverse); Mouse MMP2, 5'-GAC ATA CAT CTT TGC AGG AGA CAA G-3' (forward), 5'-TCT GCG ATG AGC TTA GGG AAA-3' (reverse); Mouse MMP9, 5'-CCT GGA ACT CAC ACG ACA TCT TC-3' (forward), 5'-TGG AAA CTC ACA CGC CAG AA-3' (reverse); Mouse TIMP1, 5'-CAT GGA AAG CCT CTG TGG ATA TG-3' (forward), 5'-GAT GTG CAA ATT TCC GTT CCT T-3' (reverse); Mouse PPAR-*α*, 5'- AGA GCC CCA TCT GTC CTC TC-3' (forward), 5'-ACT GGT AGT CTG CAA AAC CAA A-3' (reverse); Mouse 18s, 5'-AGG GGA GAG CGG GTA AGA GA-3' (forward), 5'-GGA CAG GAC TAG GCG GAA CA-3' (reverse).

Plasmid Constructs

Construction of the PPAR-α Expression Vector: The total RNA was extracted from HEK293 cells and reverse transcribed to obtain cDNA. The PPAR-α CDS secquence was acquired by PCR amplification with primers as follows: 5'-CGG CAC AAC CAG CAC CAT-3' (forward); 5'-CCA GTC CTG AGA TTA GCC ACC TAC-3' (reverse). The 1407-bp fragment were digested and ligated into pCDNA3.1 vector. Construction of the TGF-β1 promoter-pGL3 Vector: A 1348-bp fragment containing the TGF-β1 promoter was acquired by PCR amplification of genomic DNA from HEK293 cells with the following primers: 5'-TGA GTA TCA GGG AGT GGG GAA TC-3' (forward); 5'-AGG GAG GGA GCA AGC GTC-3' (reverse). The fragment was inserted upstream of a luciferase gene in the pGL3 vector (Promega). The resulting constructs were confirmed by restriction enzyme digestion and DNA sequencing.

Transfections and Reporter assays

HEK293 cells were seeded on 96-wells and transfected with 450ng/well of TGF-β1 firefly luciferase reporter plasmids or the control pGL3-Basic vector (Promega) using Lipofectamine 2000, and the renilla luciferase vector pRL-TK (50 ng/well) was cotransfected to correct for variations in transfection efficiency. 48 h after transfection, cells were harvested and lysates were analyzed for firefly and renilla luciferase activity using Dual-Luciferase Reporter Assay System (Promega) with a Lumat LB 9507 luminometer (Berthold Technologies) according to the manufacturer's instruction. The final results are represented as the fold luciferase induction compared to that of the pGL3-Basic vector.

SUPPLEMENTARY FIGURES

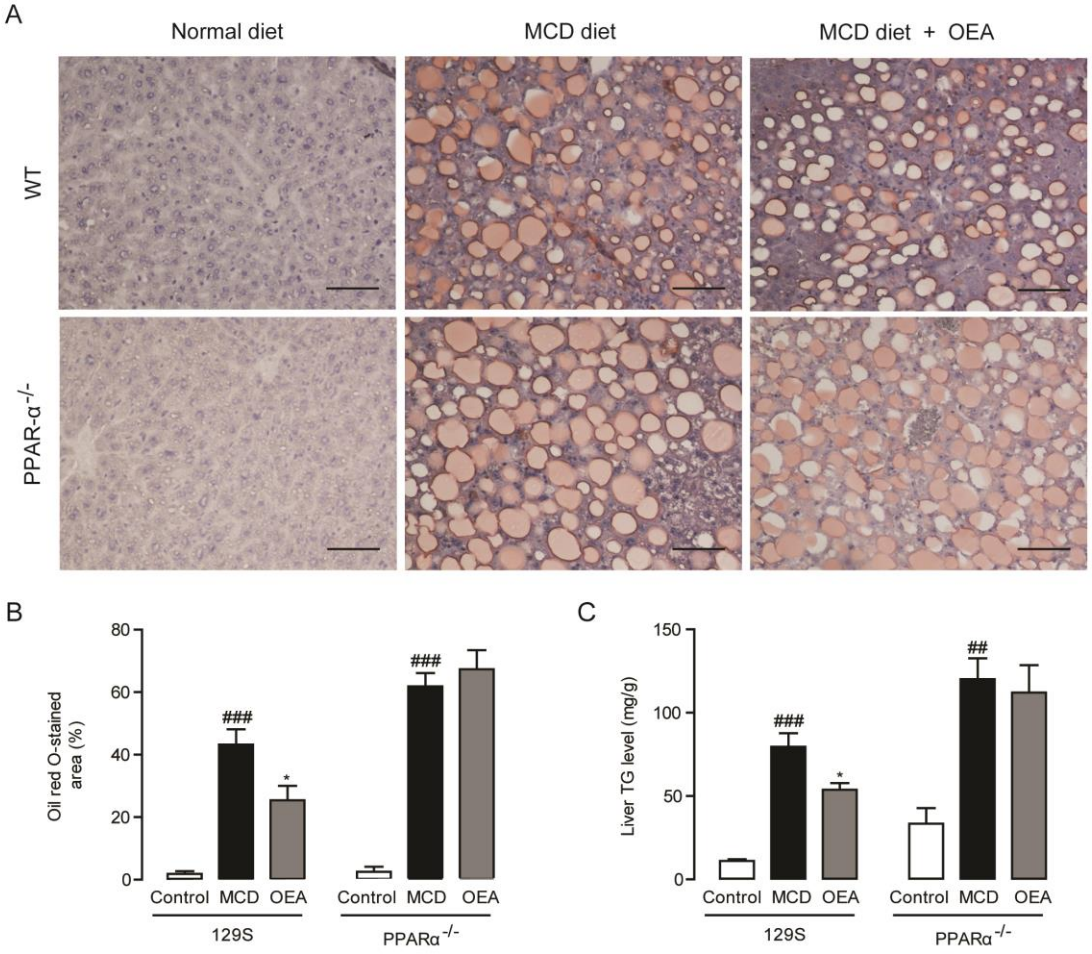

Supplementary Figure S1: OEA reduced hepatic fat storage in MCD diet-induced fibrosis mice via PPAR-*α*. (A) Oil red O staining of liver sections in wild-type (WT) mice and PPAR-*α* knockout mice fed with normal diet, MCD diet, MCD diet combined with OEA administration (5 mg/kg/day, i.p.). (B) Statistical analysis of the percentage of Oil red O-positive area in liver. (C) Hepatic TG content in wild-type (WT) mice and PPAR-*α* knockout mice fed with normal diet, MCD diet, MCD diet combined with OEA administration (5 mg/kg/day, i.p.). Data are shown as means ± s.e.m.; n = 6-8 in each group. $^{\#\#}P < 0.01$, $^{\#\#\#}P < 0.001$, $^{*}P < 0.05$. Scale bars: 100 μm.

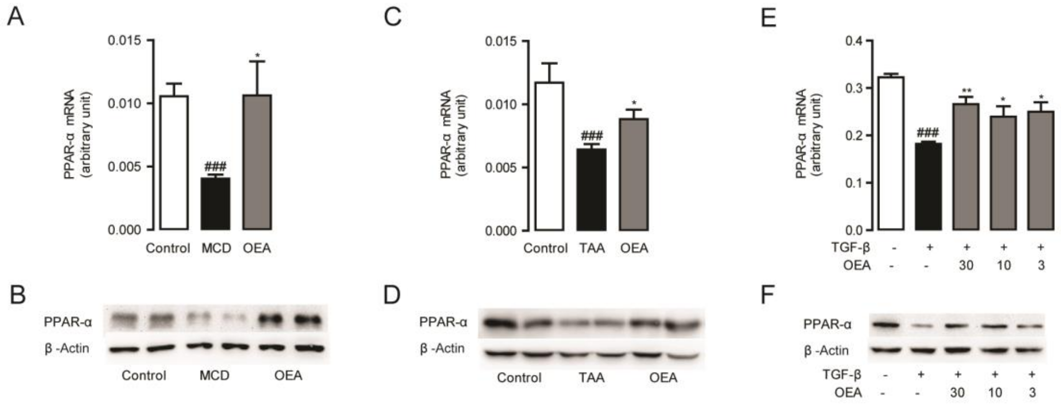

Supplementary Figure S2: The gene and protein expression of PPAR-*α* in liver samples and HSCs culture after profibrotic agents and/or OEA treatment. (A-B) Hepatic mRNA and protein expression levels of PPAR-*α* in MCD diet-induced fibrotic mice. (C-D) Hepatic mRNA and protein expression levels of PPAR-*α* in TAA-induced fibrotic mice. (E-F) The mRNA and protein expression levels of PPAR-*α* in TGF-*β*1-activated HSCs *in vitro*. Data are representative of three independent experiments each performed in duplicate assays, and values are expressed in means ± s.e.m. $^{###}$ $P < 0.001$, * $P < 0.05$, ** $P < 0.01$.

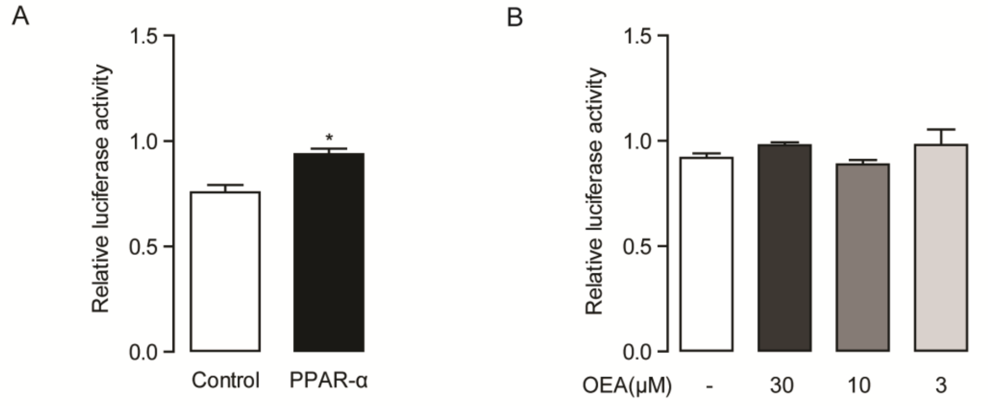

Supplementary Figure S3: Effects of PPAR-*α* overexpression and OEA treatment on TGF-*β*1 promoter activity. HEK293 cells were transfected with pGL3 vector containing the luciferase gene under the control of the the human TGF-β1 promoter plus the control reporter vector (pRL-TK). Luciferase activity levels were normalized in all cases by Renilla luciferase activity. (A) Transfections were supplemented with the indicated amount of pCDNA3.1 empty vector or pcDNA3.1 vector containing the PPAR-*α* CDS sequence. Luciferase activity was expressed as a fold change over the activity found in cells transfected with pCDNA3.1 empty vector. (B) Transfections were supplemented with OEA (30μM, 10μM, 3μM) or vehicle (DMSO). The relative fold change in luciferase activity as compared with control as shown. Data are shown as means ± s.e.m. of three independent experiments, each performed in duplicate. $^{*}P < 0.05$.

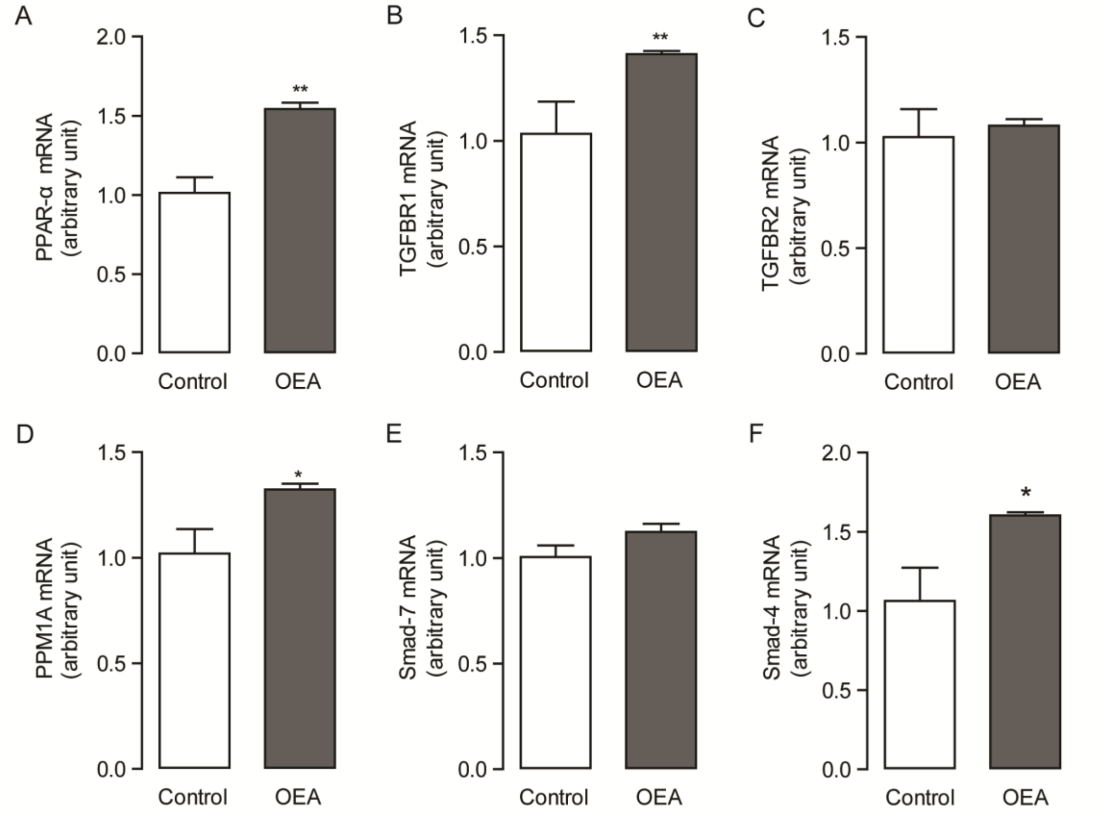

Supplementary Figure S4: The genes involved in the formation and degradation of phospho-Smad2/3 in CFSCs after OEA treatment. (A-F) CFSC cells were treated with OEA (10 μM) for 48 h, mRNA expression levels of PPAR-*α* (A), TGFBR1 (B), TGFBR2 (C), PPM1A (D), Smad7 (E), and Smad4 (F) were analyzed by real-time PCR. Data are shown as means ± s.e.m of three independent experiments, each performed in duplicate. $^{###}P < 0.001$, $^{*}P < 0.05$, $^{***}P < 0.001$.

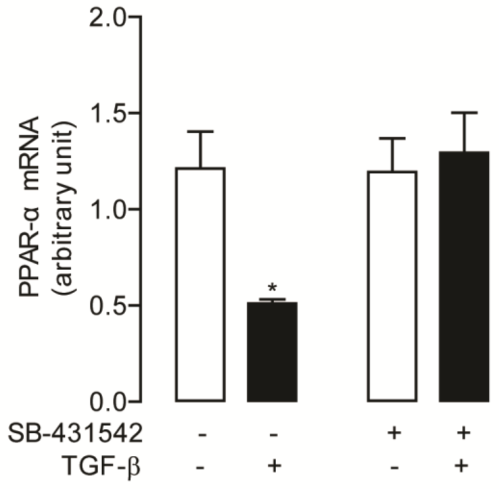

Supplementary Figure S5: TGF-β1 down-regulated PPAR-α mRNA expression *in vitro* via TGFBR1. CFSC cells were treated with TGF-β1 (5 ng/mL) for 48 h with or without TGFBR1 inhibitor SB-431542 (10 μM) treatment. The PPAR-α mRNA expression levels were measured by real-time PCR. Data are representative of three independent experiments each performed in duplicate assays, and values are expressed in means ± s.e.m. $^{*}P < 0.05$.

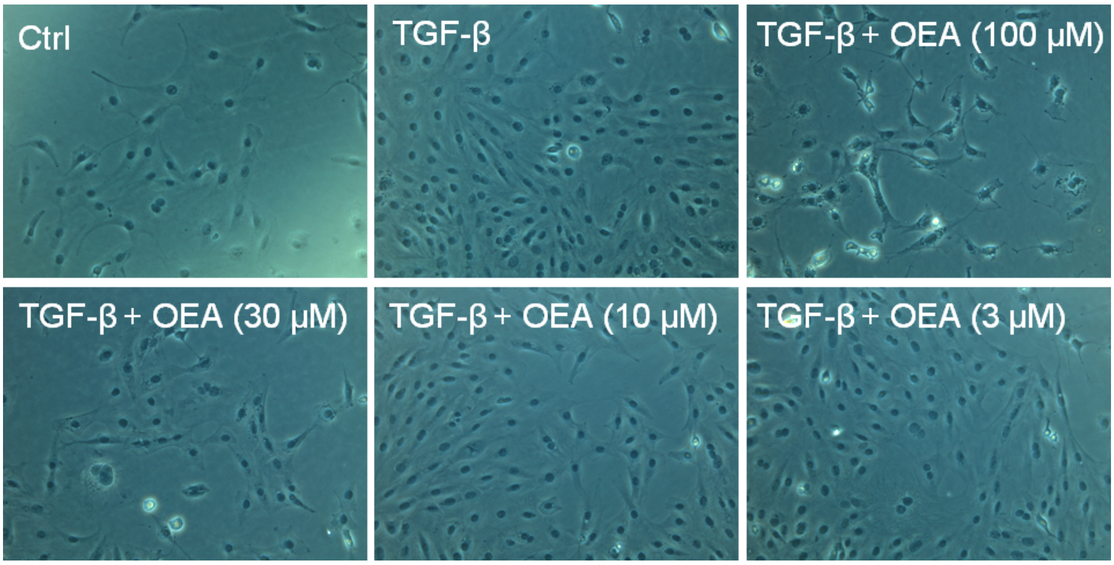

Supplementary Figure S6: OEA reversed TGF-β1 induced myofibroblastic phenotype of CFSC cells. CFSC cells were treated with OEA (100μM, 30 μM, 10 μM, 3 μM) followed by TGF-β1 (5 ng/mL) for 48 h, the morphology changes of these cells were observed under a contrast phase microscope, and photographs were taken at 200× magnification.
